# *Aegle marmelos* fruit extract attenuates *Helicobacter pylori* Lipopolysaccharide induced oxidative stress in Sprague Dawley rats

**DOI:** 10.1186/s12906-015-0915-x

**Published:** 2015-10-19

**Authors:** Yarasi Gayathri Ramakrishna, Kumarasamy Savithri, Manfred Kist, Sivasitamparam Niranjali Devaraj

**Affiliations:** Department of Biochemistry, University of Madras, Guindy Campus, Chennai, 600025 India; Institut fur Medizinische, Mekrobiologie und Hygiene, Freiburg, Germany

**Keywords:** *Helicobacter pylori* lipopolysaccharide, *Aegle marmelos*, Antioxidants, Gastric ulcer

## Abstract

**Background:**

Bael (*Aegle marmelos* (L.) Corr.) has been widely used in indigenous systems of Indian medicine to exploit its medicinal properties including astringent, antidiarrheal, antidysenteric, demulcent, antipyretic, antiulcer, anti-inflammatory and anti cancer activities. The present study aims to evaluate the antioxidative and antiulcer effect of methanolic extract of unripe fruit of *Aegle marmelos* (MEAM) against *Helicobacter pylori*-Lipopolysaccharide (HP-LPS) induced gastric ulcer in Sprague Dawley (SD) rats.

**Methods:**

Dose and duration of HP-LPS and MEAM were fixed based on ulcer index of gastric tissue of experimental animals. Various gastric secretory parameters such as volume of gastric juice, free and total acidity, acid output, pepsin concentration were analyzed. The activities of enzymatic antioxidants (superoxide dismutase, catalase, glutathione peroxidase, glutathione reductase and glutathione transferase), non-enzymatic antioxidants (reduced glutathione, vitamin C and vitamin E) and the levels of lipid peroxidation products were measured. Histological analysis was performed to evaluate the effect of *Aegle marmelos* on HP-LPS induced gastric ulcer.

**Results:**

Oral administration of HP-LPS (50 μg per animal) for four consecutive days resulted in induction of ulcer with the increase in gastric secretory parameters such as volume of gastric juice, free and total acidity, acid output, pepsin concentration. Oral administration of methanolic extract of *Aegle marmelos* fruit (MEAM) (25, 50, 100, 250 and 500 mg/kg) reduced the gastric ulcer by 2.8 %, 52.4 %, 73 %, 93 % and 93.98 %, respectively, compared to 89.2 % reduction by sucralfate (100 mg/kg). MEAM treatment significantly (*p* < 0.05) inhibited the increase in gastric secretory parameters in ulcerated rats, and it also prevented the reduction of enzymatic (superoxide dismutase, catalase, glutathione peroxidase, glutathione reductase and glutathione transferase) and non-enzymatic antioxidants (reduced glutathione, vitamin C and vitamin E) after HP-LPS induction. In addition, lipid peroxidation was inhibited by MEAM in HP-LPS induced rats. Results of histological analysis correlated well with biochemical parameters.

**Conclusion:**

These observations explored the antioxidant properties of MEAM contributing to the gastroprotective effect in HP-LPS induced gastric ulcer model.

## Background

*Helicobacter pylori* (*H. pylori*) is a highly prevalent bacterial gastroduodenal pathogen of humans infecting almost 50 % of the world’s population, and most infected individuals remain asymptomatic [1]. *H. pylori* is now recognized as a causative factor of chronic gastritis, gastroduodenal ulcer, gastric cancer and mucosa-associated lymphatic tissue lymphoma [2, 3]. Several factors of *H. pylori* mediate cytotoxicity and the inflammatory reaction. Among these are, vacuolating toxin (VacA), ammonium ions produced by urease, cytotoxin associated gene A (CagA) protein, the type IV secretion system and lipopolysaccharide (LPS) [4, 5].

In general, Gram-negative bacterial Lipopolysaccharides are key inflammation inducers. However, *Helicobacter pylori*-Lipolysaccharide (HP-LPS) shows extremely low endotoxic activity in comparison to typical Gram-negative bacterial LPS, such as those from *Escherichia coli*, which makes it to potentially contribute to the persistence of infection [6–9]. *Helicobacter pylori*, being a Gram-negative bacterium that colonizes the gastric mucosa, is recognized as a primary cause of gastric disease, and its cell-wall lipopolysaccharide has been identified among the key virulence factors responsible for eliciting mucosal inflammatory responses that characterize gastritis and duodenal ulcers [10–14]. The gastric mucosal responses associated with *H. pylori* infection in humans as well as those characterizing mucosal inflammatory changes in the animal model of HP*-*LPS induced gastritis are manifested by a marked increase in epithelial cell apoptosis and proinflammatory interleukin expression, excessive nitric oxide and prostaglandin generation, and the disturbances in NFκB and MAPK signaling cascades [15–18]. HP-LPS has been implicated in the stimulation of pepsinogen and histamine secretion, inhibition of sulfated mucin synthesis, and the production of potentially destructive auto-antibodies, which may all contribute to the loss of mucosal integrity [19]. In addition, HP-LPS was shown to bind to the gastric mucosal somatostatin receptor, thereby interfering with the regulatory effect of somatostatin on gastric mucosal G cell function [20]. Pathogen-induced inflammation is associated with an increase in Reactive Oxygen Species (ROS) and Reactive Nitrogen Species (RNS) production and the activation of the inflammatory response depletes tissue antioxidants and exposes the host to increased risk of oxidative stress [21, 22].

Eradication of *H. pylori* seems to cure both infection and ulcers. Successful treatment therefore leads to the resolution of gastritis and reduces the likelihood of ulcer recurrence [23]. The combination of a proton pump inhibitor (e.g. omeprazole) and antibiotics (i.e. ampicillin, amoxicillin, ofloxacin or tetracycline) is curative in up to 90 % of patients [24].

Many infectious diseases are known to be treated with herbal remedies throughout the history of mankind. Plant materials continue to play a major role in primary health care as therapeutic remedies in many developing countries. Plants still continue to be almost the exclusive source of drugs for the majority of the world's population. The World Health Organization reported that 80 % of the world's population rely chiefly on traditional medicine and a major part of the traditional therapies involve the use of plant extracts or their active constituents [25]. Several plants are used for the treatment of gastric ailments, including stomach ache and ulcers [26–31]. Medicinal plants are recognized as rich sources of natural antioxidants that can protect against inflammation-associated oxidative stress with their added advantages, such as less toxicity, affordability and medicinal as well as traditional values. One such traditionally used medicinal plant since ancient times is *Aegle marmelos* Correa, commonly known as Bael, which belongs to the family Rutaceae. Each and every part of the plant such as fruit, seed, leaf, bark and root is known for its therapeutic and medicinal value. The bael fruit pulp contains many functional and bioactive compounds such as carotenoids, phenolics, alkaloids, coumarins, flavonoids, terpenoids, and other antioxidants which may protect us against chronic diseases. A major constituent of the fruit is the mucilage and marmelosin (0.5 %), a coumarin. In addition, it also contains many vitamins and minerals including vitamin C, vitamin A, thiamine, riboflavin, niacin, calcium, and phosphorus [32]. It should be noted that the unripe fruits are bitter, acrid, sour, and astringent; aid digestion and stomach irritation; and are useful in treating diarrhea, dysentery, and stomachalgia [33].

Inspite of its long traditional usage in Indian medicine, antioxidant and antiulcer properties of *Aegle marmelos* fruit have not been studied *in vivo* using HP-LPS induced Sprague Dawley (SD) rats as a model. A previous study on gastric ulcer induced by aspirin in albino rats has shown the protective effect of *Aegle marmelos* fruit [34]. In a similar study, the ripe fruit pulp of AM shows gastrointestinal cytoprotective activity in Aspirin-induced, Cold-restraint stress-induced and Cerebellar lesion-induced ulcer models through the release of Serotonin (5-hydroxytryptamine; 5-HT) from enterochromaffin (EC) cells [35]. Hence, the present study was aimed to investigate the antioxidative effect of methanolic extract of unripe fruit of *Aegle marmelos* against HP-LPS induced gastric ulcer in SD rats. Secretory parameters and analyses of antioxidative enzymes were carried out using biochemical assays. These results were further confirmed by histological analysis.

## Methods

### Preparation of methanolic extract of unripe fruit of *Aegle marmelos* (MEAM)

The fruits of *A. marmelos* were collected from **Marudeeswarar** temple*,****Thiruvanmiyur,*** TamilNadu, India during the month of March 2009. The plant material was identified by Dr. Mathivanan, Professor, Centre for Advanced Study in Botany, University of Madras, Guindy Campus, Chennai, Tamilnadu, India and the specimen was deposited in the same.

The fruits were cut open, deseeded, shade dried, and ground mechanically. Dried fruit material (500 g) was first defatted with n-hexane (1 L) for 48 h. The residue was extracted with absolute methanol (1 L) for 48 h. The extract was filtered, 80 % of the solvent was removed by distillation at atmospheric pressure and the last trace of methanol was removed under reduced pressure (Yield, 21.86 %).

### Animals

Male SD rats weighing 150–200 g were obtained from the Institute of Basic Medical Science, Chennai, India. They were acclimatized to animal house conditions, fed commercial pelleted rat chow (Hindustan Lever Ltd, Bangalore, India) and had free access to water. All animals were maintained under standard conditions of temperature (28 ± 2 °C) and light (12 h light/dark cycles). The animals were housed in polypropylene cages (45 × 24 × 15 cm) and fed with standard diet pellets and water *ad libitum*. Animals were handled according to the regulations of the Institutional Animal Ethical Committee, University of Madras. All the experiments performed on animals were approved and conducted in accordance with the Institutional Animals Ethics Committee (IAEC No. 01/084/09).

### *H. pylori* Lipopolysaccharide

HP-LPS is a kind gift from Professor Manfred Kist, Institut für Medizinische Mikrobiologie und Hygiene, Freiburg, Germany. It was prepared by the conventional method from 26695 strain of *H. pylori*. The bacteria grown in Brucella broth with 5 % fetal calf serum was pelleted by centrifugation, washed twice with 0.9 % NaCl, heat inactivated for 2 h in steam and washed twice with 0.9 % NaCl. The pellet was then suspended in 1 mL 0.9 % NaCl and lyophilized. It was stored at 4^0^ C until further use.

The optimal dose of HP-LPS to induce gastric ulceration was assessed by oral administration of HP-LPS suspended in saline at different doses of 10, 25, 50, 75 and 100 μg per day for 4 consecutive days. Gastric ulcer index was measured as the sum of the length (in mm) of each lesion according to Okabe et al. [36]. The concentration of HP-LPS to induce ulcer was determined based on ulcer index of experimental animals.

### Dose determination of MEAM

Male SD rats were divided into 7 groups of six rats. Gastric ulcers were induced in all the groups by 4 days treatment with HP-LPS (50 *μ*g per animal per day). Groups 2 to 6 were treated orally with different doses of MEAM dissolved in water (25, 50, 100, 250 and 500 mg per kg body weight) for 10 days after ulcer induction with HP-LPS. Group 7 rats, which served as reference control, were treated with Sucralfate (100 mgkg^−1^ dissolved in water and administered orally) for 10 days [37].

After the experimental period, the rats of all the groups were sacrificed by cervical decapitation. The stomachs were removed and cut open along the greater curvature and then examined under a light microscope. Based on the ulcer index, effective dose of treatment was determined.

Further work was carried out with the grouping of animals as below:

- Group I: Control
- Group II: Induced (HP-LPS alone [50 *μ*g per animal per day])
- Group III: Treated (HP-LPS 50 *μ*g per animal per day + 250 mg of MEAM per kg body weight.
- Group IV: Reference control (HP-LPS 50 *μ*g per animal per day + 100 mg of Sucralfate per kg body weight.
- Group V: Drug control (250 mg of MEAM per kg body weight)

### Pyloric ligation

Pyloric ligation was done by the method of Shay Komarov [38]. After the experimental period, rats were subjected to pyloric ligation under ether anaesthesia for the collection of gastric juice. Under light anaesthesia, the abdomen was opened by a small midline incision below the xiphoid process; the pyloric portion of the stomach was slightly lifted out and ligated, avoiding damage to its blood supply. The stomach was replaced and the abdominal wall was closed by interrupted sutures.

### Determination of acid secretory parameters

The animals were sacrificed 4 h after pylorus ligation. Stomach was dissected out, cut open and the gastric juice was drained into a small beaker, and centrifuged at 2000 rpm for 10 min. The supernatant was collected and used for the estimation of volume of gastric juice, pH, free acidity, total acidity, pepsin concentration and acid output. The volume was noted and expressed as ml/100 g/4 h and pH was measured using pH meter. Estimation of free and total acidity in gastric juice was carried out by the method of Card and Marks [39]. Free acidity and total acidity were determined by titrating with 0.01 N sodium hydroxide using Toepfer’s reagent and phenolphthalein as indicator, respectively. The acidity was expressed as mEq/l/100 g and acid output as mEq/100 g/4 h. Pepsin was assayed according to the method of Anson [40] using haemoglobin as substrate. The absorbance of the solution was read at 650 nm. Results were expressed as μmol of tyrosine liberated/ml.

### Preparation of gastric tissue

The stomach was excised, rinsed in ice-cold physiological saline and homogenized in 0.1 M Tris–HCl buffer (pH 7.4), using a tissue homogenizer with a teflon pestle, at 4 °C. The resultant tissue homogenate was used for biochemical measurements.

### Biochemical analysis

Total protein was estimated by the method of Lowry et al. [41]. Enzymatic antioxidants were measured in the gastric tissues. SOD was measured by the method described by Misra and Fridovich [42]. CAT and GPx were measured according to Takahara et al. [43] and Rotruck et al. [44], respectively. Glutathione reductase (GR) was analyzed by the method of Staal et al. [45]. Non-enzymatic antioxidants, namely Vitamin E, C and reduced glutathione (GSH) were measured by the method of Desai [46], Omaye et al. [47] and Ellman [48], respectively. Glutathione-S-transferase activity was assayed by the method of Habig et al. [49]. LPO was determined in the gastric tissue by measuring the formation of thiobarbituric acid reactive substances (TBARS) according to the method of Ohkawa et al. [50].

### Histological studies

Gastric tissues were initially rinsed with ice-cold 0.9 % saline to remove the debris adhering to tissues. The tissues were then fixed in 10 % buffered formalin, routinely processed and embedded in paraffin. Sections (5 mm thick) were cut and stained with haematoxylin and eosin. The slides were then evaluated under light microscope (Nikon XDS-1B).

### Statistical methods

All the grouped data were evaluated using SPSS/10.0 software. Hypothesis testing method included one-way analysis of variance (ANOVA) followed by least significant difference (LSD) test. *p <* 0.05 was considered to indicate statistical significance. All the results were expressed as mean ± S.D. for six rats in each group.

## Results

### Dose determination of HP-LPS

**Fig. 1:**
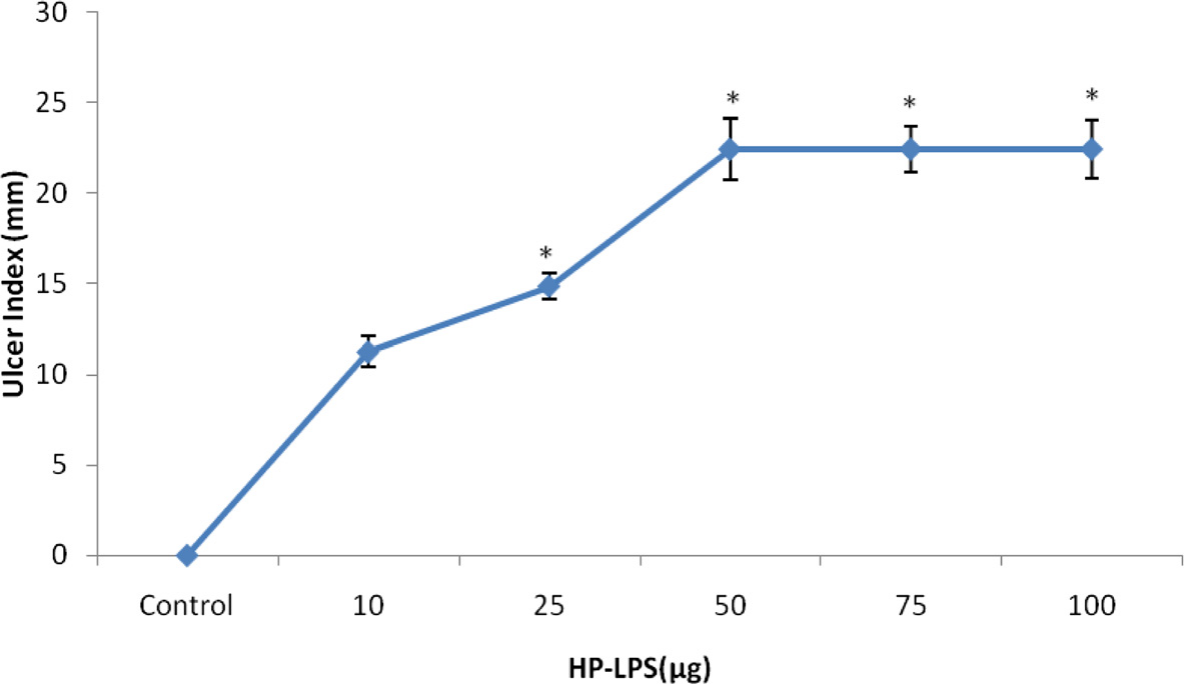
Dose determination of HP-LPS for the induction of gastric lesions in SD rats. Data are expressed as Mean ± SD for six animals in each group. Statistical significance: *p < 0.001, all groups vs control

Figure 1 depicts the dose determination curve of HP-LPS to induce gastric ulcer in SD rats. Doses of 10 and 25 μg of HP-LPS per day for a period of 4 consecutive days produced slight increase in ulcer index when compared with control, whereas it tends to increase (*p* < 0.001) drastically with 50, 75 and 100 μg per day for the same period in a dose dependent manner. Hence, 50 μg of HP-LPS per day for four days was considered as the minimum effective dose to induce gastric ulcer in SD rats.

### Dose determination of MEAM

**Fig. 2:**
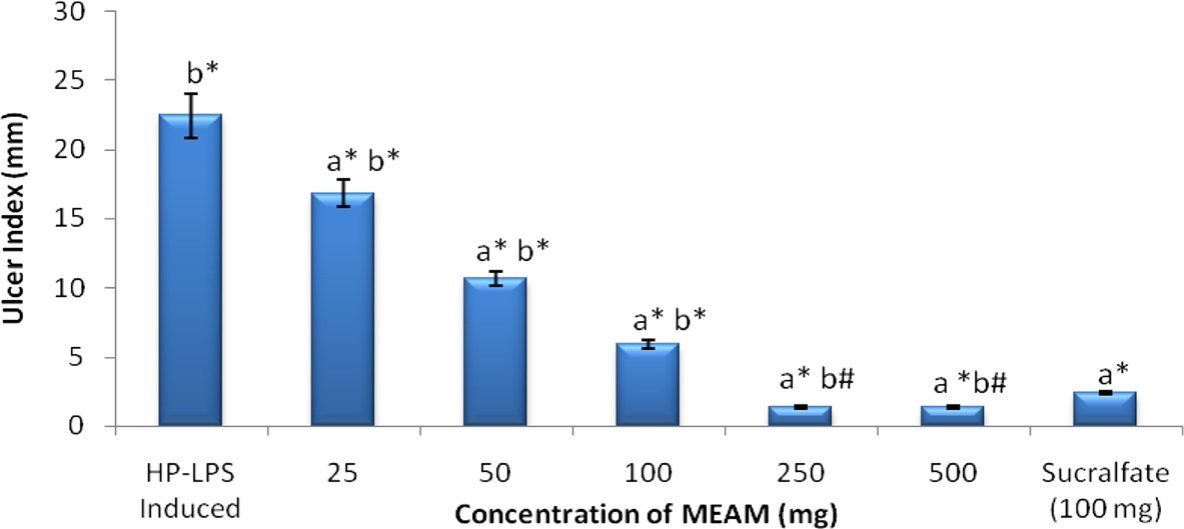
Effective dose determination of Methanolic extract of *Aegle marmelos* (MEAM) in HP-LPS induced animals. Data are expressed as Mean ± SD for six animals in each group. Statistical significance: *p < 0.001; #*p* <0.05; NS, not significant. a: all groups vs HP-LPS induced; b: all groups vs rats treated with sucralfate

Effective dose determination of MEAM treatment against HP-LPS-induced gastric ulcers is shown in Fig. 2. Oral administration of MEAM (25, 50, 100, 250 and 500 mg/kg) reduced the gastric ulcer by 2.8 %, 52.4 %, 73 %, 93 % and 93.98 %, respectively, compared to 89.2 % reduction by sucralfate (100 mg/kg). There was a significant dose-dependent decrease *(p* < 0.001) in the ulcer index of rats treated with MEAM compared with untreated ulcerated rats. Therefore, 250 mg/kg of MEAM for 10 days was chosen as the minimum effective dose of MEAM which offered significant protection. Hence, this dose was used for further evaluation of the gastroprotective activity of MEAM.

### Effect of MEAM on secretory parameters

MEAM significantly inhibited the increase in secretory parameters as shown in Table 1. In ulcerated rats (Group II), there was significant increase (*p* < 0.05) in volume of gastric juice, free acidity, total acidity, acid output and pepsin concentration when compared with that of control. However, these parameters were restored to normal levels upon MEAM treatment (Group III), similar to that of control animals (Group I). Sucralfate treated animals (IV) also produced similar results. Drug alone administered animals (Group V) did not show any significant changes in comparison to that of control animals (Group I).

**Table 1:** Effect of MEAM on the levels of acid secretory parameters in gastric juice of experimental rats

Groups	Volume of Gastric Juice	pH	Free acidity	Total acidity	Acid Output	Pepsin concentration
I	1.45 ± 0.10	4.6 ± 0.28	32.69 ± 1.93	58.73 ± 3.95	85.16 ± 5.74	155.13 ± 10.24
II	3.27 ± 0.27^a^*	1.92 ± 0.14^a^*	60.17 ± 4.39^a^*	91.82 ± 5.22^a^*	300.25 ± 21.03^a^*	262.91 ± 17.85^a^*
III	1.65 ± 0.12^b^*	4.45 ± 0.35^b^*	33.26 ± 2.28^b^*	59.97 ± 3.47^b^*	98.95 ± 7.27^b^*	174.36 ± 13.03^b^*
IV	1.7 ± 0.11^b^*	4.2 ± 0.27^b^*	33.9 ± 2.26^b^*	60.66 ± 4.15^b^*	103.12 ± 7.59^b^*	180.88 ± 8.87^b^*
V	1.4 ± 0.10^c NS^	4.7 ± 0.34^c NS^	32.41 ± 2.22^c NS^	58.11 ± 4.65^c NS^	81.35 ± 5.45^c NS^	155.18 ± 10.20^c NS^

Data are expressed as Mean ± S.D. for six animals in each group. Units: volume of gastric juice (ml 100 g^−1^ 4 h^−1^); free acidity (mEq L^−1^100 g^−1^); total acidity (mEq^−1^ L 100 g^−1^); acid output (mEq 100 g^−1^ 4 h^−1^); pepsin activity (*μ*mol tyrosine liberated mL^−1^). Statistical significance: *p < 0.05; NS, not significant. a: Group II compared with Group I. b: Groups III and IV compared with Group II. c: Group V compared with Group I

### Effect of MEAM on enzymatic antioxidants and non enzymatic antioxidants

The differences in the activities of enzymatic antioxidants and in the levels of non-enzymatic antioxidants are summarized in Tables 2 and 3, respectively. Upon HP-LPS induction, a significant decrease (*p* < 0.05) in activities of enzymatic antioxidants (SOD, CAT, GPx, GR and GST) was observed in Group II animals when compared with that of control animals (Group I). MEAM treatment to ulcerated rats significantly elevated the activities of these enzymes to near normal levels. Highly significant reduction (*p* < 0.05) in the levels of non enzymatic antioxidants (GSH, vitamin E and vitamin C) was evident in the HP-LPS induced animals (Group II). These adverse changes were reversed to normalcy in Group III (MEAM treated) animals. Group IV and V animals did not show any significant changes when compared to control animals.

**Table 2:** Effect of MEAM on the activities of enzymatic antioxidants in experimental animals

Groups	SOD	CAT	GPx	GR	GST
I	4.43 ± 0.26	17.63 ± 0.91	206.28 ± 14.49	2.64 ± 0.16	4.82 ± 0.24
II	2.51 ± 0.18^a*^	9.06 ± 0.61^a*^	138.28 ± 37.39^a*^	1.39 ± 0.11^a*^	3.02 ± 0.19^a*^
III	4.39 ± 0.30^b*^	17.32 ± 1.15^b*^	205.74 ± 15.07^b*^	2.51 ± 0.15^b*^	4.8 ± 0.38^b*^
IV	4.41 ± 0.28^b*^	17.47 ± 1.24^b*^	204.93 ± 13.40^b*^	2.62 ± 0.18^b*^	4.79 ± 0.26^b*^
V	4.42 ± 0.20^c NS^	17.58 ± 1.12^c NS^	205.62 ± 12.67^c NS^	2.63 ± 0.22^c NS^	4.81 ± 0.35^c NS^

Data are expressed as Mean ± SD for six animals in each group. Units: SOD, Units/mg protein (one unit of the SOD activity is the amount of enzyme required to give 50 % inhibition of epinephrine auto oxidation); CAT, μmol of H_2_O_2_ consumed/min/mg protein; GSH, nmol/g tissue; GPx, nmol GSH oxidized/min/mg protein; GR, μmol NADPH oxidized/min/mg protein; GST, μmol of 1-chloro-2,4 dinitrobenzene conjugate formed/min/mg protein. Statistical significance: *p < 0.05; NS, not significant. a: Group II (HP-LPS induced) compared with Group I (Control). b: Groups III (MEAM treated)and IV (Sucralfate treated) compared with Group II (HP-LPS induced). c: Group V (Drug control) compared with Group I (Control)

**Table 3:** Effect of MEAM on the activities of non-enzymatic antioxidants in experimental animals

Groups	GSH	Vit C	Vit E
I	3.92 ± 0.23	7.52 ± 0.33	5.85 ± 0.32
II	1.46 ± 0.10^a*^	4.64 ± 0.36^a*^	3.08 ± 0.21^a*^
III	3.89 ± 0.25^b*^	7.48 ± 0.55^b*^	5.8 ± 0.44^b*^
IV	3.8 ± 0.25^b*^	7.45 ± 0.46^b*^	5.75 ± 0.37^b*^
V	3.88 ± 0.33^c NS^	7.5 ± 0.54^c NS^	5.79 ± 0.34^c NS^

Data are expressed as Mean ± S.D. for six animals in each group. Units: GSH, μg/mg protein; Vitamin E, μg/mg protein; Vitamin C, μg/mg protein. Statistical significance: *p < 0.05; NS, not significant. a: Group II (HP-LPS induced) compared with Group I (Control). b: Groups III (MEAM treated)and IV (Sucralfate treated) compared with Group II (HP-LPS induced). c: Group V (Drug control) compared with Group I (Control)

### Effect of MEAM on lipid peroxidation

**Fig. 3:**
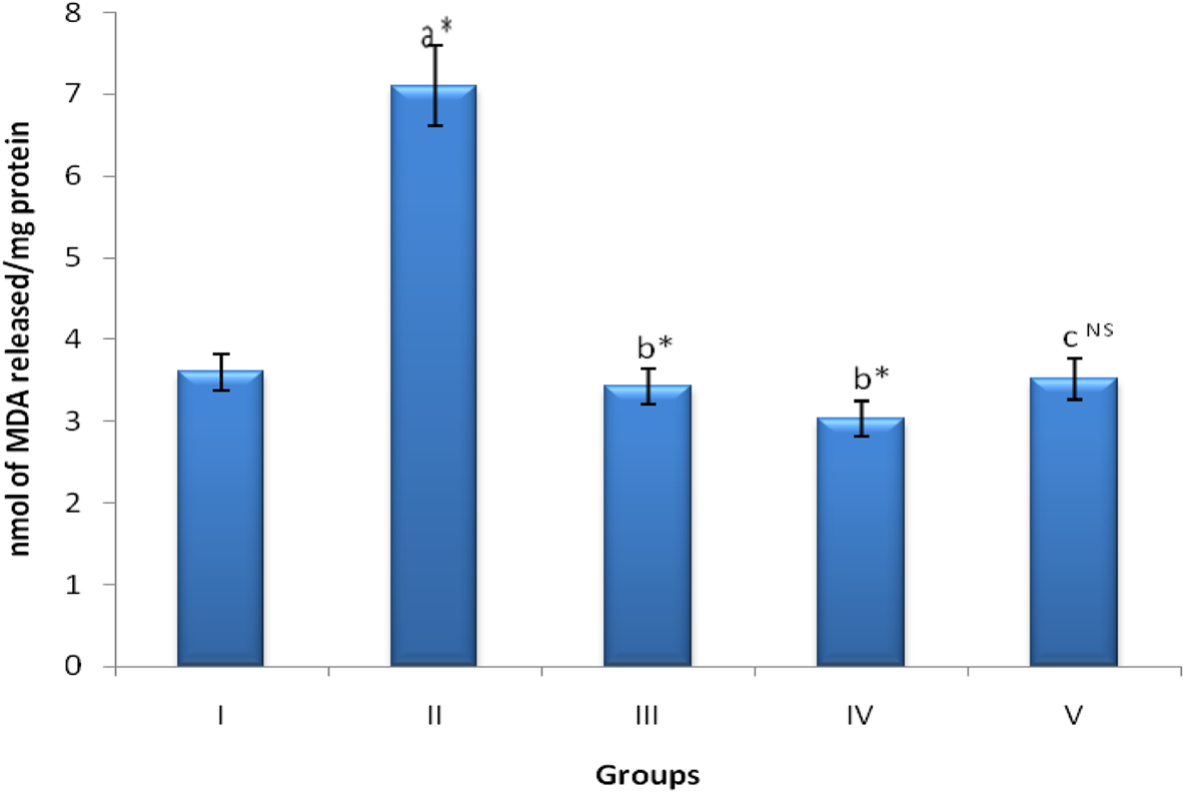
Effect of MEAM on Lipid Peroxidation in Gastric tissue of Experimental Animals. Data are expressed as Mean ± SD for six animals in each group. Units: LPO, nmol of MDA released/mg protein. Statistical significance: *p < 0.05; NS, not significant. a: Group II (HP-LPS induced) compared with Group I (Control). b: Groups III (MEAM treated)and IV (Sucralfate treated) compared with Group II (HP-LPS induced). c: Group V (Drug control) compared with Group I (Control)

Figure 3 depicts the extent of lipid peroxidation in gastric tissue of control and experimental groups of rats. A remarkable elevation in the levels of TBARS was observed in the HP-LPS induced rats when compared to control rats. Treatment with MEAM resulted in a significant decrease (*p* < 0.05) in these levels when compared to ulcerated animals. However, MEAM alone and sucralfate treated animals did not show any significant changes in these levels when compared to that of control animals.

### Histological analysis of gastric mucosa

**Fig. 4:**
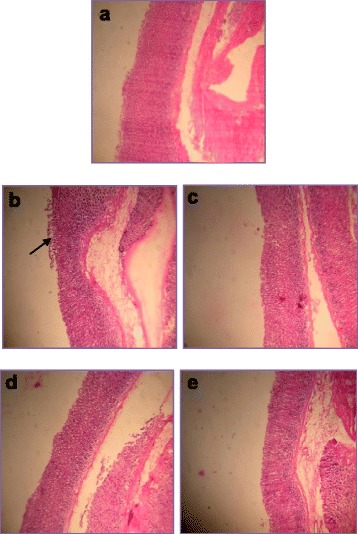
Histological analysis of gastric mucosa of control and experimental animals. Sections stained with Hematoxylin and Eosin were visualized under light microscope. **a** Control gastric mucosa shows normal architecture of epithelial lining **b** Gastric mucosa of rats induced with HP-LPS shows marked inflammation, neutrophil infiltration and mucosal ulceration with sub-mucosal edema **c** Gastric mucosa of MEAM treated rats shows no ulcers, mucosal regeneration with mild inflammatory cells, and mild edema. **d** Gastric mucosa of rats treated with Sucralfate also shows similar architecture as that of MEAM **e** Gastric mucosa of rats treated with MEAM alone shows normal gastric epithelial lining similar to that of control. Magnification: 5 X

The effect of MEAM on gastric histology of control and experimental group of animals is depicted in Fig. 4. The gastric mucosa of control rats showed normal architecture with perfect intact structures and regular epithelial lining (Fig. 4a). The gastric mucosa of HP-LPS induced rats showed ulceration, submucosal oedema, inflammation and polymorphonuclear infiltration at the ulcer site (Fig. 4b); whereas, the gastric mucosa of rats treated with MEAM showed no ulceration, instead exhibited regenerating epithelium with mild inflammation (Fig. 4c). However, the gastric mucosa of rats treated with sucralfate also showed slight regeneration of superficial epithelium and with less inflammatory cells (Fig. 4d). The gastric mucosa of drug control animals showed normal architecture similar to that of control animals (Fig. 4e).

## Discussion

Though various pharmacological interventions, alone, or in combination, have proved to be beneficial in treatment of gastric ulcer in several studies, they are not free from their adverse side effects. These detrimental effects have triggered immense interest in a search for alternative herbal medicines. Hence, this study was aimed to find the effect of methanolic extract of unripe fruit of *Aegle marmelos* against HP-LPS induced gastric ulcer in SD rats.

In this study, a reduction in the ulcer index was noticed in MEAM treated group of animals which clearly points towards the beneficiary effects of the extract. High gastric acid production in patients significantly develops antrum predominant gastritis and is at increased risk for duodenal ulcers. Low gastric acid secretion, in contrast, develops chronic atrophic gastritis and carcinoma [51]. *H. pylori* infection in patients results in significant increase in acid output and duodenal ulceration, indicating a key role of acid in the pathogenesis of gastro mucosal ulceration. Highly acidic pH (low pH) has been shown to enhance *H.pylori* induced NF-κB nuclear binding [52]. Increased acid secretions by HP-LPS contributes to mucosal damage of stomach. At glandular level, HP-LPS can stimulate histamine release from rat enterochromaffin cells which contributes to increase in acid secretion [53]. Pathological effects produced by HP-LPS on gastric mucosa were varied depending on the strain of the bacterium, which, in turn, depends on the molecular structure of the LPS preparation. HP-LPS in its pure form obtained from a known pathological strain can stimulate acid secretion and pepsinogen [54]. The present study indicated the stimulated levels of acid secretion by HP-LPS, confirming the role in gastric ulceration. *H. pylori* LPS has also been implicated in a variety of other putative mechanisms of gastric damage that are less well characterized and require independent verification. These include effects on mucus glycosylation, sulfation and epithelial cell interaction and acid secretion [53–57].

Gastric acid secretion can be beneficial while it acts as a part of the host defense mechanism preventing bacterial infection and hence pathogenicity. It has been proposed that the local acid production will determine the extent of colonization by *Helicobacter* species in the stomach, with decreased acid production promoting the spread of colonization to the acid-producing body of the stomach as opposed to the increased acid production, which tends to restrict colonization, predominantly to the gastric antrum [58]. LPS from *H. pylori* 26695 also stimulated acid secretion as it is evident in this study, confirming its role in gastric mucosal damage. However, on treatment with MEAM, acid secretion was significantly reduced, suggesting its antisecretory role.

Pepsin, a protease present in the gastric lumen, plays a crucial role in ulceration of the stomach and, in fact, gastric acid does not cause ulceration in the absence of pepsin. Pepsinogen, an inactive precursor secreted by the chief cells of the gastric mucosa, is activated by acid in the gastric lumen. *H. pylori* LPS has been implicated in the stimulation of pepsinogen and histamine secretion in association with gastric mucosal damage [10] and the results of the present study coincide with these findings. The anti-secretory effect of MEAM was manifested by the attenuation of acid secretory parameters – acidity, acid output and pepsin activity.

Sucralfate, an ammonium salt of sucrose octasulfate has been shown to prevent the formation of acute gastric lesions induced by various ulcerogens in experimental animals [59]. The potency of Sucralfate in healing and reducing the gastroduodenal ulceration was supported by several clinical trials [60]. The mechanisms by which Sucralfate shows the protective and antiulcer effects were not fully explained but they have been attributed to the binding of the drug to the defective and ulcerated mucosa [61, 62], the formation of a protective barrier over the eroded mucosal surface and decrease of pepsin [63, 64]. It is recently reported that Sucralfate may also stimulate the luminal release of prostaglandins, which contribute to the protective and ulcer healing properties of drug [65]. Sucralfate markedly suppresses *H. pylori* infection and the accompanying hypersecretion of acid. These effects are likely to be important mechanisms by which the drug promotes healing of duodenal ulcers [66]. Hence, sucralfate was used as reference control.

An important feature of the pathogenesis of *H. pylori* infection is its persistence in the inflamed gastric mucosa progressing towards peptic ulceration and gastric carcinoma. *H. pylori* is believed to be a major aetiological agent that causes chronic gastritis, along with the other features, including the lymphoid follicles or lymphoid aggregates, surface epithelial degradation with mucous depletion, and intestinal metaplasia. One characteristic event in gastritis is an infiltration of the sub-epithelial gastric lamina propria by phagocytes, mainly neutrophils and macrophages, which produce large amounts of ROS (reactive oxygen species) in the host defence reaction [67–69]. There is evidence that *H. pylori* infection leads to increased production of O_2_^−^ via NADPH oxidase in gastric cells, stimulated by lipopolysaccharide as well as xanthine oxidase, another mechanism for the generation of oxygen-derived free radicals [70, 71]. The oxygen radicals, which are produced in gastric epithelial cells infected with *H. pylori*, may reduce the antioxidant defense mechanism and turn on the expression of inflammatory genes, adhesion molecules and mediators stimulating cell proliferation, as well as defensive molecular chaperones in gastric epithelial cells [72].

It is evident that an oral administration of *H. pylori* LPS can trigger distinct inflammatory responses in rat gastric mucosa [44]. ROS are believed to be involved in inflammation, the expression of oncogenes and cell proliferation [68]. These mediators impart oxidative stress on the gastric epithelium in the immediate vicinity.

Normally, the level of oxidative stress is regulated by antioxidants, including vitamin C, cellular reduced glutathione etc., but levels of these antioxidants are decreased during infection. The increased levels of pro-oxidative factors and decreased levels of antioxidants result in severe oxidative stress that can modulate many processes in the gastric epithelium [73]. Cells can survive against chronic oxidative stress by enhancing activities of antioxidant enzymes, thereby protecting cells from DNA damages. The results of the present study demonstrate that MEAM strongly increases both the enzymatic and nonenzymatic antioxidants.

Pharmacological effects of the medicinal plants are well known for their free-radical scavenging properties. The bael fruit pulp contains many functional and bioactive compounds such as carotenoids, phenolics, alkaloids, coumarins, flavonoids, terpenoids, and other antioxidants which protect us against chronic diseases. Total dietary fiber found in this fruit is divided into insoluble dietary and soluble dietary fiber (mucilage and pectin). In addition, it also contains many vitamins and minerals including vitamin C, vitamin A, thiamine, riboflavin, niacin, calcium, and phosphorus [74–76]. Therefore, bael fruit is one of the important plants used for indigenous traditional medicine [77–80], and it is evident from the above study that *Aegle marmelos* (Bael) fruit, containing various active constituents, with their synergistic activities, proves effective against HP-LPS induced ulcer.

## Conclusion

In conclusion, *A. marmelos* fruit extract shows good antioxidant activity. Hence, it exerts its antiulcer effect by inhibiting secretory parameters and activating antioxidant mechanism, thus protecting the gastric mucosa against HP-LPS induced ulceration.
